# Biosynthesis of a water solubility‐enhanced succinyl glucoside derivative of luteolin and its neuroprotective effect

**DOI:** 10.1111/1751-7915.14095

**Published:** 2022-06-21

**Authors:** Liangliang Chen, Siyuan Chang, Lin Zhao, Bingfeng Li, Sen Zhang, Chenke Yun, Xiao Wu, Jingyi Meng, Guoqing Li, Sheng Guo, Jinao Duan

**Affiliations:** ^1^ Jiangsu Key Laboratory for High Technology Research of TCM Formulae, Jiangsu Collaborative Innovation Center of Chinese Medicinal Resources Industrialization Nanjing University of Chinese Medicine 138 Xianlin Road Nanjing 210023 Jiangsu China; ^2^ College of Life and Health Nanjing Polytechnic Institute 625 Geguan Road Nanjing 210048 Jiangsu China

## Abstract

The natural flavonoids luteolin and luteoloside have anti‐bacterial, anti‐inflammatory, anti‐oxidant, anti‐tumour, hypolipidemic, cholesterol lowering and neuroprotective effects, but their poor water solubility limits their application in industrial production and the pharmaceutical industry. In this study, luteolin‐7‐*O*‐β‐(6″‐*O*‐succinyl)‐d‐glucoside, a new compound that was prepared by succinyl glycosylation of luteolin by the organic solvent tolerant bacterium *Bacillus amyloliquefaciens* FJ18 in an 8.0% DMSO (v/v) system, was obtained and identified. Its greater water solubility (2293 times that of luteolin and 12 232 times that of luteoloside) provides the solution to the application problems of luteolin and luteoloside. The conversion rate of luteolin (1.0 g l^−1^) was almost 100% at 24 h, while the yield of luteolin‐7‐*O*‐β‐(6″‐*O*‐succinyl)‐d‐glucoside reached 76.2%. In experiments involving the oxygen glucose deprivation/reoxygenation injury model of mouse hippocampal neuron cells, the cell viability was significantly improved with luteolin‐7‐*O*‐β‐(6″‐*O*‐succinyl)‐d‐glucoside dosing, and the expressions of the anti‐oxidant enzyme HO‐1 in the nucleus increased, providing a neuroprotective effect for ischemic cerebral cells. The availability of biosynthetic luteolin‐7‐*O*‐β‐(6″‐*O*‐succinyl)‐d‐glucoside, which is expected to replace luteolin and luteoloside, would effectively expand the clinical application value of luteolin derivatives.

## Graphical abstract

Luteolin‐7‐O‐β‐(6″‐O‐succinyl)‐d‐glucoside's high water solubility (2293 times of luteolin and 12 232 times of luteoloside) perfectly solved the application problem of luteolin and luteoloside. Luteolin‐7‐O‐β‐(6″‐O‐succinyl)‐d‐glucoside had the neuroprotective effect on cerebral ischemic cells. Biosynthesis of luteolin‐7‐O‐β‐(6″‐O‐succinyl)‐d‐glucoside, which is expected to replace luteolin and luteoloside, would effectively expand the clinical application value of luteolin derivatives.

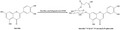

## Introduction

Luteolin, a natural flavonoid, is widely found in plants (e.g. *Lonicera japonica* Thunb., *Dendranthema morifolium* (Ramat.) Tzvelev, *Perilla frutescens* (L.) Britt., *Nepeta cataria* L.), which are often used as traditional Chinese medicines to treat diseases. It has a variety of pharmacological properties, such as anti‐bacterial (Laban *et al*., 2003), anti‐inflammatory (Zhu *et al*., 2011; Xie *et al*., 2021), anti‐oxidation (Rooban *et al*., 2012; Assunção *et al*., 2021) and anti‐tumour (Attoub *et al*., 2011; Xie *et al*., 2012) activities. At present, with the whole world experiencing COVID‐19, only luteolin and abyssinone II, out of 43 flavonoids from seven different classes, might act as potential therapeutic candidates for SARS‐CoV‐2 infection because of their good antiviral activities (Shawan *et al*., 2021). However, like other natural flavonoid aglycones, luteolin also has a small dipole moment and low molecular polarity and is therefore only slightly soluble in water, which limits its application in the pharmaceutical industry and industrial production.

Glycosylation, as a means of improving the water solubility of compounds, is often used to modify the water solubility of natural products, including flavonoids. And it has been found that the glycosylation of hydroxyl groups has no adverse effect on the anti‐oxidant activity of the natural flavonoids. Quercetin‐3‐*O*‐glucoside has better water solubility compared with quercetin due to glycosyl conjugation (Makino *et al*., 2013), while it also has a higher bioavailability (Valentova *et al*., 2014). The water solubility of daidzin is 6 times that of daidzein, and it was found that daidzin has greater bioavailability than daidzein during oral dosing in humans (Rüfer *et al*., 2008). The glycosidic sites of luteolin are the 3′‐, 4′‐ and 7‐hydroxyl groups, which form luteolin‐3′‐*O*‐glucoside (Kim *et al*., 2007), luteolin‐4′‐*O*‐glucoside (Ko *et al*., 2006) and luteolin‐7‐*O*‐glucoside (Ko *et al*., 2006). Among these, luteolin‐7‐*O*‐glucoside, or luteoloside, retains physiological activities that are similar to those of luteolin, such as strong bactericidal and anti‐inflammatory properties, as well as good anti‐oxidation, anti‐virus and anti‐tumour effects (Stefano *et al*., 2021), but its water solubility is unexpectedly lower than that of luteolin, which means that glycosylation modification alone cannot bring about the efficient utilization of luteolin derivatives.

Succinylation is a common method of protein structure modification. Compared with methylation and acetylation, it can lead to changes in protein properties. In the molecular modification of natural products, it is expected that more and better physiologically active substances will be found by combining glucosylation with succinylation. Toda *et al*. (1999) and Park *et al*. (2010) reported that *Bacillus* exhibits succinylation activity towards flavonoid glycosides. Chang *et al*. (2018) pointed out that 6‐succinylglucoside should be formed by succinylation of the corresponding glucoside. At the same time, they used *Bacillus subtilis* ATCC 6633 to biotransform the triterpenoid compound antcin K, and achieved the synthesis of 25*S*‐antcin K 26‐*O*‐β‐(6′‐*O*‐succinyl)‐glucoside and 25*R*‐antcin K 26‐*O*‐(6′‐*O*‐succinyl)‐glucoside.

Since Zaks and Klibanov (1988) found that a lipase can survive in organic solvents and retain high thermal stability and catalytic activity, a nonaqueous biocatalysis system has developed rapidly that could effectively solve the problem of poor solubility of compound substrates in water. Wu *et al*. (2013) screened several strains from more than 600 oil contaminated soil samples, among which the β‐glucosidase expressed by *Arthrobacter nicotianae* XM6 could transform puerarin into fructose‐β‐(2, 6)‐puerarin in 25% dimethyl sulfoxide (DMSO). Compared with an aqueous reaction system, the concentration of puerarin could be increased more than 11‐fold, and the yield was as high as 91%. In the preliminary work of our team, the efficient glycosylation of luteolin was accomplished by the *Lonicera japonica* rhizosphere strain *Myroides odoratimimus* XT02 in 15% DMSO (v/v) organic medium with a much higher conversion rate of luteolin than that in water (Zhao *et al*., 2018). In this study, *Bacillus amyloliquefaciens* FJ18, an organic solvent tolerant bacterium, was chosen to realize the succinyl glycosylation modification of luteolin in a nonaqueous system. The main succinyl glycosylation product, a new compound, was separated and purified, and its structure was identified by ultra‐performance liquid chromatography‐QToF/MS (UPLC‐QToF/MS) and nuclear magnetic resonance (NMR) analysis. Its higher water solubility than luteolin was confirmed, and its protective mechanism against oxygen glucose deprivation/reoxygenation (OGD/R) injury of mouse hippocampal neuron (HT22) cells was explored preliminarily, which could open up new applications of luteolin derivatives.

## Results and discussion

### Biosynthesis of luteolin derivatives

**Fig. 1:**
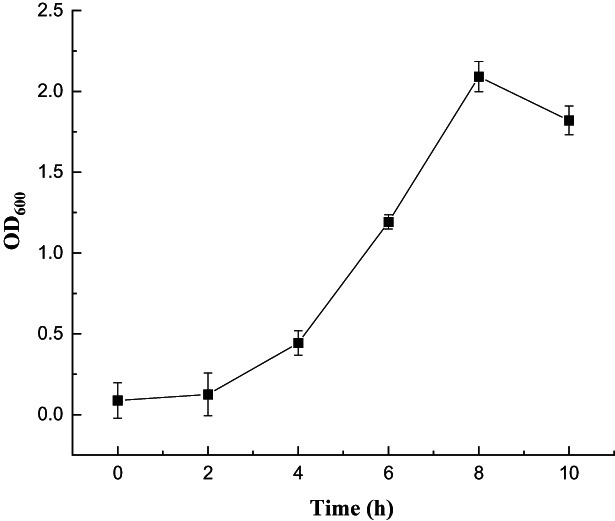
Growth curve of *Bacillus amyloliquefaciens* FJ18.

**Fig. 2:**
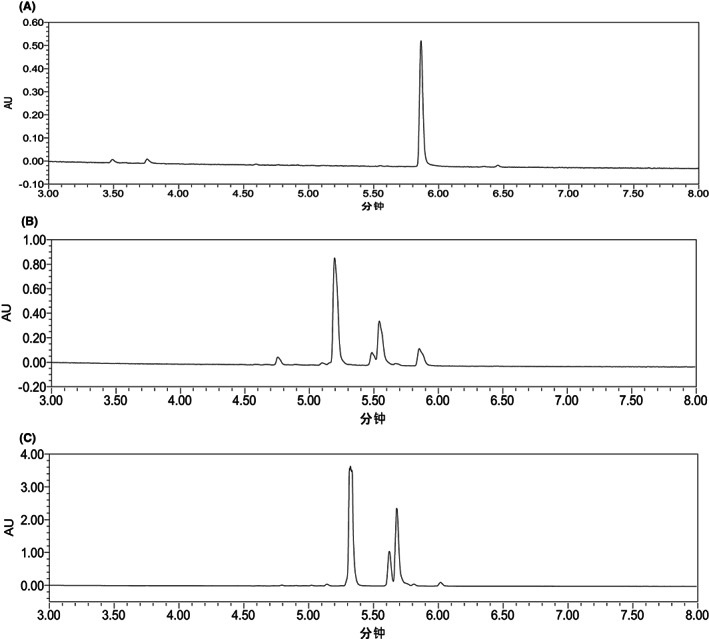
UPLC analysis of the biotransformation of luteolin by *Bacillus amyloliquefaciens* FJ18. (A–C) 0, 12 and 24 h, respectively.

During the fermentation, the growth curve of *Bacillus amyloliquefaciens* FJ18 displayed a logarithmic growth phase after 2 h, while the stable phase was at 8–10 h, which was determined to be the optimal fermentation termination point (Fig. 1). The UPLC analysis of the biotransformation of luteolin by the fermented microorganism is shown in Fig. 2. In addition to luteolin (5.935 min), there were another three peaks (5.261, 5.549 and 5.610 min), representing different luteolin derivatives. It is worth noting that in a nonaqueous system (8.0% DMSO), the biotransformation of luteolin (1.0 g l^−1^) at a gram level was successfully realized again in this work. The conversion rate of luteolin was almost 100% at 24 h, while the yield of the main product reached 76.2%, which was different from our previous study of the biosynthesis of luteoloside by *Myroides odoratimimus* (a solvent‐tolerant bacterium from the rhizosphere of *Lonicera japonica*) (Zhao *et al*., 2018). In terms of biological modification alone, this is a major innovation in microbial catalytic systems.

The emergence of organic solvent resistant microorganisms and their organic solvent resistant enzymes has provided a new way to solve the core problem of utilizing an enzyme in a non‐aqueous phase biotransformation reaction (Zaks and Klibanov, 1988). Therefore, the high stabilities of strains in organic solvents are the basis of non‐aqueous enzymatic biotransformation and high efficiency biocatalysis.

### Purification of luteolin derivatives

Macroporous adsorption resin is an active material with a huge surface area. It can achieve the separation and purification of a substance and removal of impurities under certain conditions, depending on the force between it and the adsorbent material and the molecular weight of the adsorbed molecule (Zhang and Li, 2013). However, due to the similar physical properties of the conversion products in this work, the requirements of the elution conditions for complete separation of the products by macroporous adsorption resin were greater. In order to achieve efficient separation of the products, a middle chromatogram gel (MCI GEL) material, which is a high separation efficiency medium pressure mass spectrometric separation gel that is widely used in the separation of fermentation products, was used for further separation.

The mixture (Fig. 2C) produced by the biotransformation of luteolin was well separated by the macroporous adsorption resin AB‐8 and the MCI GEL packing column, and the purities of samples (solid) reached 98% according to the UPLC analysis (Fig. S1). The structure of the main product in the biotransformation, product 1 (5.217 min), was identified by UPLC‐QTof/MS analysis and NMR analysis.

### Identification of the luteolin succinoside derivative

The protonated form ([M‐H]‐) of product 1 occurred at 547.1069 m/z, and this molecular ion produced a new ion at 447.0934 m/z according to UPLC‐QToF/MS (Fig. S2). Based on the molecular mass of luteolin (286.2390), it was speculated that one glycosl group was connected to luteolin forming luteoloside (447.0934 m/z) and a succinyl group was connected to luteoloside forming a luteolin succinyl glucoside derivative (547.1069 m/z, product 1).

**Fig. 3:**
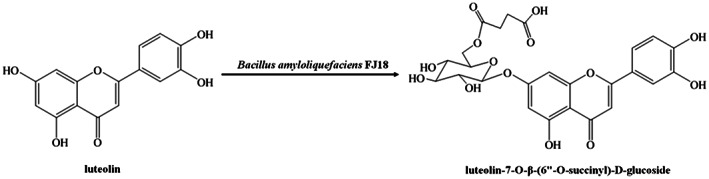
Biosynthesis of luteolin‐7‐*O*‐β‐(6″‐*O*‐succinyl)‐d‐glucoside from luteolin with *Bacillus amyloliquefaciens* FJ18.

In order to further verify our deduction, 10 mg of purified product 1 (solid) was sent to Jiangsu Simcere Pharmaceutical Co. Ltd., Jiangsu, China, and analysed by NMR (Figs S3–S6). The relative molecular mass of product 1 was determined to be 547.1069, and its structure was identified as luteolin‐7‐*O*‐β‐(6″‐*O*‐succinyl)‐d‐glucoside (7‐SGL, Fig. 3), a new luteolin succinyl glucoside derivative. This is the first time a 7‐position succinoside derivative of luteolin has been obtained with high efficiency by microbial fermentation, and it has great research value due to its pharmacological activity and potential industrial applications.

### Greater water solubility of 7‐SGL compared to luteolin

Luteolin has many pharmacological activities, but its clinical application is greatly limited because of its poor water solubility and low bioavailability. The water solubility of luteolin is 0.0064 mg ml^−1^ (Table S1), and that of luteoloside is only 0.0012 mg ml^−1^ (Table S2), indicating that glycosylation alone could not improve its affinity to water. However, further succinylation gave unexpected results. The water solubility of 7‐SGL is 14.6782 mg ml^−1^ (30°C), 2293 times greater than luteolin and 12 232 times greater than luteolin glycoside (Tables 1 and S3).

**Table 1:** Water solubilities of luteolin and luteolin derivatives.

Sample	Water solubility (mg ml^−1^)	Multiple (compared with luteolin)	Multiple (compared with luteolin glycoside)
Luteolin	0.0064	–	–
Luteolin glycoside	0.0012	–	–
7‐SGL	14.6782	2293	12 232

In the practical applications of natural active ingredients, their water solubilities greatly affect the metabolic processes they undergo in the body and the development and utilization of related products. In particular in the field of medicine, in order to improve the efficacy of a natural product, blindly increasing the dosage carries the risk of toxic accumulation of active ingredients. Moreover, drugs with poor water solubility are not readily formulated as intravenous injections and oral liquids (Lipinski *et al*., 2001), which greatly limits the development of their pharmaceutical preparations. Luteolin, a highly active compound, also faces this problem in practical application. In this study, the results showed that the water solubility of luteolin and luteolin glycoside could be greatly improved by using *Bacillus amyloliquefaciens* FJ18 to modify luteolin. And the improvement in water solubility is closely related to the extensive use of luteolin, luteolin glycoside resources and natural drugs with these substances as the main effective components, which is a significant enhancement of the industrial application value of these components.

### Protective mechanism of 7‐SGL on OGD/R injury of HT22 cells

**Fig. 4:**
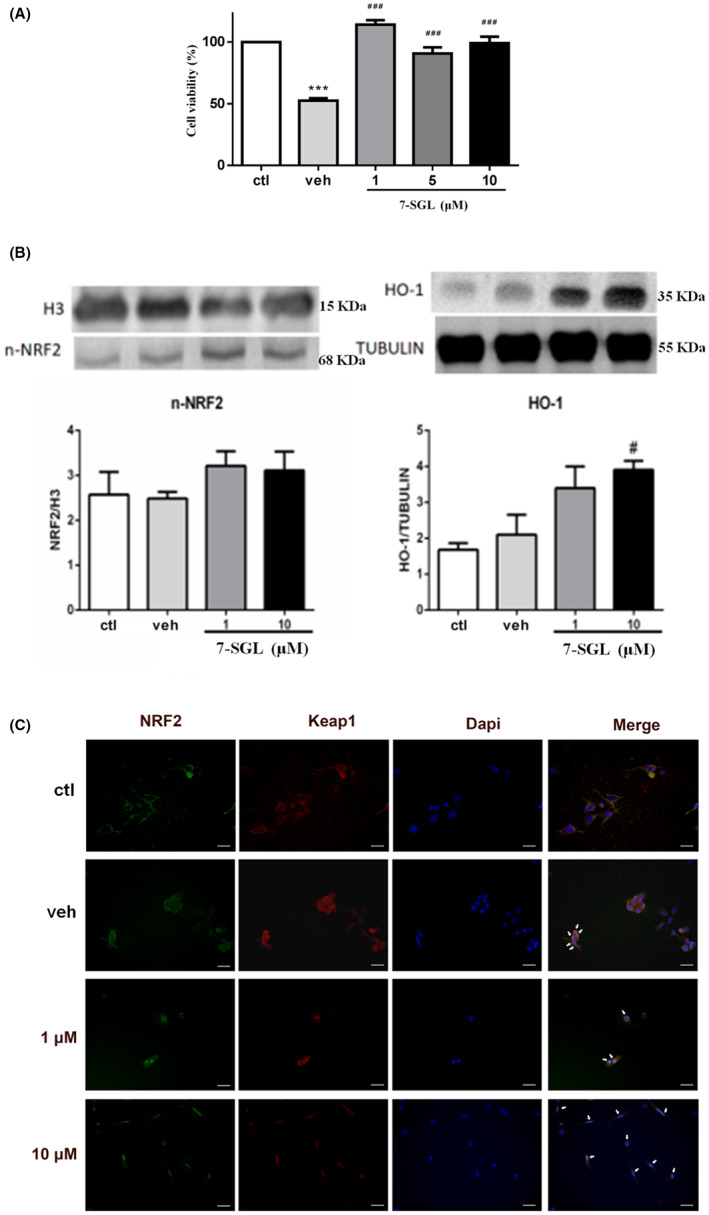
7‐SGL regulates the NRF2/HO‐1 pathway in H22 cells. A. 7‐SGL protect HT22 cells from ODG/R injury. B. Increased level HO‐1 induced by 7‐SGL. NRF2 in nucleus (n‐NRF2) revealed no significant difference among groups. (C) Treated groups showed obvious translocation of NRF2 (green) from cytoplasm to the nucleus (blue), while Keap1 (red) remained in cytoplasm, as revealed by immunofluorescence (scale bar = 20 μm). ctl, normal group; veh, OGD/R group; 1 (or 1 μM), 5 and 10 (or 10 μM), drug groups with 1 μM, 5 μM and 10 μM of 7‐SGL dosing, respectively. H3 and TUBULIN, loading control. *P < 0.05, **P < 0.01, ***P < 0.001, compared with ctl. #*P* < 0.05, ##*P* < 0.01, ###*P* < 0.001, compared with veh. [Colour figure can be viewed at wileyonlinelibrary.com]

In the MTT assay, the cell viability of HT22 cells in the OGD/R group was only half (*P* < 0.001, vs. ctl) that of the normal group, which showed the successful establishment of the model. Upon the intervention of 7‐SGL, the cell viability of HT22 cells in the three drug groups was significantly improved (*P* < 0.001, vs. veh), and was similar to the normal group. The effect of the low‐dose group (1 μM) was the most obvious (Fig. 4A).

The OGD/R injury model is widely used in the study of injury of isolated level cerebral ischemia–reperfusion (Luo *et al*., 2013; Wei *et al*., 2018). During the ischemic cell reoxygenation and glucose reperfusion, mitochondria mediate the production of a large number of reactive oxyradicals (related to reactive oxygen species, ROS), which leads to the aggravation of oxidative damage (Luo *et al*., 2013). However, interference by an anti‐oxidant could significantly inhibit the increase in ROS and reduce the oxidative stress response related to mitochondria. The inducible enzyme HO‐1 can consume free oxygen and reduce the production and accumulation of ROS, which would reduce the degree of cell damage (Li *et al*., 2016). Therefore, in order to study whether the expression of HO‐1 protein in cells increased and reflected the neuroprotective effect in the cerebral ischemic cells under 7‐SGL intervention, we performed western blot analysis of nuclear protein expression. The results showed that HO‐1 levels were significantly increased (10 μM, *P* < 0.05, vs. veh) following 7‐SGL administration; however, there was no significantly difference of NRF2 in nucleus (n‐NRF2) levels among the groups (Fig. 4B). So, 7‐SGL could increase the level of HO‐1 to achieve the neuroprotective effect in the cerebral ischemic cells (Barone *et al*., 2014; Chao *et al*., 2014), although it was unclear from this data whether NRF2 was involved in this pathway. And in the results of cell immunofluorescence staining, 7‐SGL promoted nuclear translocation of NRF2 compared with the control group (Fig. 4C).

In the normal physiological state, most molecules of the transcription factors NRF2 and Keap1 are combined and inactivated (Baird and Yamamoto, 2020), and cytoplasmic NRF2 cannot enter into the nucleus to activate HO‐1 (Guo *et al*., 2015; Suzuki and Yamamoto, 2015). But under external stimulation, NRF2/Keap1 complex disintegrate and are transferred to the nucleus where they participate in the transcription of a series of enzyme genes, thus promoting the expression of anti‐oxidant enzymes (inducing HO‐1) and effectively clearing the excess oxygen‐free radicals produced in the body (Li *et al*., 2013; Zenkov *et al*., 2013).

Luteolin is known to have anti‐oxidant capacity (Rooban *et al*., 2012; Assunção *et al*., 2021). In this study, whether the luteolin succinoside derivative 7‐SGL has a similar pharmacological activity or not was also explored. The results showed that 7‐SGL could improve the survival rate of OGD/R injury model cells, and increase the level of the inducible enzyme HO‐1 (might be related to promoting nuclear translocation of NRF2) showing an anti‐oxidant mechanism.

In the current study, luteolin‐7‐*O*‐β‐(6″‐*O*‐succinyl)‐d‐glucoside, a new luteolin glycoside derivative (yield of 76.2%), was bio‐synthesized by the biotransformation of luteolin (1.0 g l^−1^) in the fermentation broth of *Bacillus amyloliquefaciens* FJ18 in a nonaqueous system and was separated using the macroporous adsorption resin AB‐8 and an MCI GEL packing column, and its structure was identified. It had better water solubility than luteolin (2293 times), and could exert a neuroprotective effect on ischemic cerebral cells by increasing the expression of the anti‐oxidant enzyme HO‐1.

## Experimental procedures

### Materials

*Bacillus amyloliquefaciens* FJ18 was obtained and preserved from nature by our team, and its China Centre for Type Culture Collection (CCTCC) No. is M 2016272. Luteolin (98%) and luteoloside (98%) were purchased from Nanjing Qingyun Biotechnology, Nanjing, China.

### Microbial fermentation

*Bacillus amyloliquefaciens* FJ18 (stored at −80°C) was transferred to a Luria‐Bertani (LB)‐agar plate (10 g l^−1^ tryptone, 5 g l^−1^ yeast extract, 10 g l^−1^ NaCl, 20 g l^−1^ agar) at 30°C for 24 h. Next, the reactivated strain was incubated in 100 ml of LB media at 30°C with shaking at 180 rpm for 10–12 h. Then, 2 ml of the initial culture were inoculated into 100 ml of fresh MLBRT medium (8 g l^−1^ tryptone, 4 g l^−1^ yeast extract, 3 g l^−1^ KH_2_PO_4_, 6 g l^−1^ sucrose, 0.5 g l^−1^ MnSO_4_, pH 7.2–7.5 (adjusted with 4 mM NaOH)) in 500‐ml flasks at 30°C and 180 rpm for another 8–10 h. During the fermentation, the microbial growth state was measured by the turbidimetric method. A little microbial solution was removed every 2 h and the OD_600_ value was measured with a UV visible spectrophotometer (UV‐5100; Shanghai Yuanxi Instrument, Shanghai, China).

### Biological modification of luteolin

After the fermentation, 84 ml of the fermentation broth and 16 ml of a DMSO solution of luteolin (200 mg) were added to 100 ml of phosphate buffer (pH 7.4, 9.59 g l^−1^ Na_2_HPO_4_, 1.74 g l^−1^ KH_2_PO_4_), and the mixture was shaken at 180 rpm and 30°C for 24 h for the microbial transformation. During incubation, samples (1 ml) were removed at defined time points and clarified by centrifugation at 13 523 *g* for 5 min. Next, the supernatant was mixed with an equal volume of methanol, clarified by centrifugation at 13 523 *g* for another 2 min, and then analysed immediately by UPLC or stored at −20°C.

### Extraction of luteolin derivatives

The reaction mixture catalysed by the fermentation broth of *Bacillus amyloliquefaciens* FJ18 was clarified by centrifugation at 13 523 *g* for 10 min, diluted 2–3 times with acetic acid solution (pH 4.0) and added to the adsorption macroporous resin AB‐8 (particle size 0.30–1.25 mm, 10 times the volume of the sample), which was soaked in anhydrous ethanol for 24 h in advance and washed with acetic acid solution until ethanol was removed. Next, another 4–5 times the resin volume of acetic acid solution was used to wash the adsorption macroporous resin to remove the impurities (sucrose, DMSO and so on). Then, the preliminary purified sample was washed with methanol and collected. The full flow rate was 1 ml min^−1^.

An MCI GEL packing column was used for further sample refining. The pretreatment of the fillers and the loading steps were the same as above. The preliminary purified sample was diluted 4–5 times with acetic acid solution and added to the column. Then, 3 times the resin volume of different concentrations of methanol aqueous solutions (50%, 55% and 60%) were used to wash the MCI GEL packing in order of increasing concentration, and the collecting solutions were separated every 40–50 ml and analysed immediately by UPLC. The full flow rate was also 1 ml min^−1^.

After the column separation, the collected solutions were first evaporated to remove methanol, frozen completely in the refrigerator (−80°C), and then dried in the freeze dryer to obtain the purified products.

### Water solubility of luteolin and luteolin derivatives

Determination of the luteolin standard curve: the luteolin mother liquor (1 g l^−1^) was prepared by accurately weighing 10 mg of luteolin in a 10‐ml volumetric flask and diluting with methanol to the marker. Then, different volumes of mother liquor (0.2, 0.4, 0.8, 1.2 and 1.6 ml) were transferred to new 10‐ml volumetric flasks and diluted with methanol again to obtain different concentrations of luteolin standard solutions. These samples were analysed by UPLC, and their concentrations were linear regressed by the peak areas.

Preparation of the test solutions: because of the low solubilities of luteolin and luteoloside, 800 μl of the supernatant of a supersaturated aqueous solution of luteolin (or luteoloside) were dried, redissolved in 80 μl of methanol and analysed by UPLC, while 10 μl of the supernatant of a supersaturated aqueous solution of luteolin succinyl glucoside derivative were diluted with methanol to 1000 μl at different temperatures and analysed by UPLC.

### Establishment of the OGD/R model

HT22 cells were cultured to the logarithmic growth stage, their density was adjusted to 4 × 10^4^ ml^−1^, and 100 μl per well were inoculated on 96 well plates and cultured in a 5% CO_2_ incubator at 37°C until the cells adhered to the wall. After discarding the supernatant, the cells were washed twice with serum‐free Earle's balanced salt solution (EBSS), resuspended with 100 μl of serum‐free EBSS and incubated in a three gas (5% CO_2_, 0.5% O_2_, 94.5% N_2_) incubator for 2 h to create oxygen glucose deprivation (OGD). Finally, the cells were resuspended with normal medium after removing the supernatant and cultured in a 5% CO_2_ incubator at 37°C for 6 h to establish the OGD/R injury model (Luo *et al*., 2013).

### MTT assay

The HT22 cells were randomly divided into three groups: a model group (OGD/R), a normal group (NA) and a drug group. According to the results of pre‐experiment screening, the drug group consisted of three concentrations of active small molecule administration (1, 5 and 10 μM), which occurred at 24 h before and during modelling. The model group and drug group were established according to the method in ‘*Establishment of OGD/R model*’, while the normal group was established by culturing the cells in the normal medium in a 5% CO_2_ incubator at 37°C. After the OGD/R injury model was established, 20 μl of MTT (5 mg ml^−1^) were added to the cells in each well of the plate, incubated in a 5% CO_2_ incubator (in the dark) at 37°C for 4 h, resuspended with 150 μl DMSO after removing the supernatant and dissolved by shaking at low speed for 20 min. The absorbance values of the samples (A) were detected at 492 nm by a microplate reader. In this study, a circle within the 96‐well plate was filled with sterile PBS to prevent an edge effect. A blank control group was treated in the same manner as the normal group, except without cells.

$$\text{Cell viability}\;\left(\%\right)=\left(\text{model group}\;A\;\text{or drug group}\;A-\text{blank control group}\;A\right)/\left(\text{normal group}\;A-\text{blank control group}\;A\right)*100\%$$

where model group A, normal group A, drug group A and blank control group A represent the values of the model group, normal group, drug group and blank control group, respectively.

### Western blot

The nuclear protein, extracted from HT22 cells by a nuclear protein extraction kit, was determined by the BCA method. Samples (20 μg each) were separated by 10% sodium dodecyl sulfate‐polyacrylamide gel electrophoresis (SDS‐PAGE), transferred to a PVDF membrane, sealed with 1% bovine albumin (BSA) for 1 h, incubated at 4°C overnight with different primary antibodies added, and incubated at room temperature for 1 h with secondary antibody added; images were then collected by a tanon chemiluminescence imaging system after adding electrochemiluminescence (ECL). We used the following antibodies in western blotting: rabbit anti‐HO‐1 (ab13243, 1:1000, from Abcam, Shanghai, China), mouse anti‐NRF2 (ab89443, 1:500, from Abcam), rabbit anti‐TUBULIN (10068‐1‐AP, 1:3000, from Proteintech, Shanghai, China), rabbit anti‐histone H3 (17168‐1‐AP, 1:1000, from Proteintech).

### Immunofluorescence staining

After a period of cell culture in a 96‐well plate, the climbing plate was taken out, and the blocking solution containing goat serum and Triton X‐100 was added for 1 h. Next, the cells were incubated at 4°C overnight with different primary antibodies added, rewarmed for 1 h and rinsed with PBS buffer 3 times (5 min each time). The corresponding fluorescent secondary antibody was dripped into the solution, then the cells were incubated at 37°C for another 1 h, rinsed with PBS buffer 3 times (5 min each time) and used for a 15‐min photophobic reaction with Hoechst solution (5 μg ml^−1^) added. After termination of the reaction, the cells were rinsed with PBS buffer several times, and the expression (fluorescence intensity) of the corresponding protein was observed with a fluorescence microscope. The fluorescence intensity of the protein was analysed by Image‐Pro software. We used the following antibodies in immunofluorescence: rabbit anti‐Keap1 (ab139729, 1:1000, from Abcam), and mouse anti‐NRF2 (ab89443, 1:500, from Abcam).

### UPLC analysis

UPLC analysis was conducted on a Waters Acquit UPLC I‐Class (Waters Technology (Shanghai), Shanghai, China) equipped with a Waters 2996 PDA monitor (wavelength = 210–400 nm) and a Waters ACQUITY UPLC® BEH C18 column (100 mm × 2.1 mm, 1.7 μm). The column temperature and flow rate were 30°C and 0.4 ml min^−1^, respectively. Mobile phase A was water with 0.1% formic acid added, and mobile phase B was acetonitrile. Gradient elution: 0.00–2.00 min, 95% A; 2.00–12.00 min, 95%–5% A; 12.00–13.00 min, 5% A; 13.00–13.50 min, 5%–95% A; 13.50–16.00 min, 95%–5% A. The injection volume was 2 μl (Stefano *et al*., 2021).

To determine the solubilities of the samples, UPLC analysis was conducted on a Waters Acquit UPLC I‐Class equipped with a Waters 2996 PDA monitor (wavelength = 210–400 nm) and an Alltima C18 column (250 mm × 4.6 mm, 5 μm). The column temperature and flow rate were 30°C and 1.0 ml min^−1^, respectively. Mobile phase A was water with 0.1% formic acid added, and mobile phase B was methanol. Gradient elution: 0.00–20.00 min, 90%–5% A; 20.00–23.50 min, 5% A; 23.50–27.00 min, 5%–90% A; 27.00–35.00 min, 90% A. The injection volume was 10 μl.

### UPLC‐QToF/MS analysis

UPLC‐QToF/MS analysis was conducted on a Waters Acquit UPLC I‐Class system coupled with a XevoTM G2 QToF (Waters MS Technologies, Manchester, UK), a quadrupole and orthogonal acceleration time‐of‐flight tandem mass spectrometer. Mass spectra in negative ion mode were acquired under the following conditions: collision energy voltage = 6 eV, ion source temp = 120°C, desolvent temp = 350°C, desolvent gas flow = 900 l h^−1^ (nitrogen), capillary voltage = 3000 V, cone voltage = 30 V, m/z range = 100–1000. A Phenomenex Luna Omega C18 column (2.1 × 100 mm, 1.6 μm) was used and maintained at 30°C, with UV detection at 260 nm. The flow rate was 0.4 ml min^−1^, using 0.1% formic acid in water (A) and acetonitrile (B) as the mobile phase. The UPLC elution conditions were: 0.00–8.00 min, 90%–5% A; 8.00–9.00 min, 5% A; 9.00–9.10 min, 5%–90% A; 9.10–10.00 min, 90% A. The injection volume was 2 μl.

### Statistical analysis

In this work, SPSS 18.0 statistical software was used for data processing, and the results are expressed as $\bar{x}\pm s$. Comparisons between two groups were made by the *t*‐test, while comparisons among groups were made by analysis of variance. The differences between groups were considered to be statistically significant when *P* < 0.05.

## Author contributions

LC, SC, SZ, XW and JD conceived and designed the study. LC, SC, LZ, CY, JM and GL performed the experiments and analysed the data. LC wrote the manuscript. SC, BL, SZ, XW, SG and JD reviewed and edited the manuscript. All authors read and approved the manuscript.

## Conflict of interest

The authors declare that they have no competing interests.

## Supporting information


**Fig. S1.** UPLC analysis of the purified luteolin derivatives. (A) product 1, retention time = 5.217 min; (B) product 2, retention time = 5.505 min; (C) product 3, retention time = 5.587 min.
**Fig. S2.** UPLC‐QToF/MS analysis of product 1.
**Fig. S3.** ^1^H NMR spectrum of 7‐SGL.
**Fig. S4.** ^13^C NMR spectrum of 7‐SGL.
**Fig. S5.** HMBC NMR spectrum of 7‐SGL.
**Fig. S6.** HSQC NMR spectrum of 7‐SGL.
**Fig. S7.** Standard curve for determination of water solubility of luteolin.
**Fig. S8.** Standard curve for determination of water solubility of luteoloside.
**Fig. S9.** Standard curve for determination of water solubility of 7‐SGL.
**Fig. S10.** Water solubility of 7‐SGL at different temperatures.
**Table S1.** Solubility determination of luteolin at 30°C.
**Table S2.** Solubility determination of luteoloside at 30°C.
**Table S3.** Solubility determination of 7‐SGL at different temperatures.
