# Supplementary material for: Biosynthesis of a water solubility‐enhanced succinyl glucoside derivative of luteolin and its neuroprotective effect
Source: Microb Biotechnol. 2022 Jun 21;15(9):2401–10. doi: 10.1111/1751-7915.14095 (PMC9437877; doi:10.1111/1751-7915.14095)
Supplement: Supplementary file 1 — Fig. S1. UPLC analysis of the purified luteolin derivatives. (A) product 1, retention time = 5.217 min; (B) product 2, retention time = 5.505 min; (C) product 3, retention time = 5.587 min. Fig. S2. UPLC‐QToF/MS analysis of product 1. Fig. S3. 1H NMR spectrum of 7‐SGL. Fig. S4. 13C NMR spectrum of 7‐SGL. Fig. S5. HMBC NMR spectrum of 7‐SGL. Fig. S6. HSQC NMR spectrum of 7‐SGL. Fig. S7. Standard curve for determination of water solubility of luteolin. Fig. S8. Standard curve for determination of water solubility of luteoloside. Fig. S9. Standard curve for determination of water solubility of 7‐SGL. Fig. S10. Water solubility of 7‐SGL at different temperatures. Table S1. Solubility determination of luteolin at 30°C. Table S2. Solubility determination of luteoloside at 30°C. Table S3. Solubility determination of 7‐SGL at different temperatures. [file MBT2-15-2401-s001.docx]

**Supporting Information**

**Biosynthesis of a water solubility-enhanced succinyl glucoside derivative of luteolin and its neuroprotective effect**

Liangliang Chen^1#^, Siyuan Chang^2#^, Lin Zhao^1^, Bingfeng Li^2^, Sen Zhang^1^*, Chenke Yun^1^, Xiao Wu^1^*, Jingyi Meng^1^, Guoqing Li^1^, Sheng Guo^1^, Jinao Duan^1^

*^1^ Jiangsu Collaborative Innovation Center of Chinese Medicinal Resources Industrialization, Jiangsu Key Laboratory for High Technology Research of TCM Formulae, Nanjing University of Chinese Medicine, 138 Xianlin Road, Nanjing 210023, Jiangsu, China*

*^2^ College of Life and Health, Nanjing Polytechnic Institute, 625 Geguan Road, Nanjing 210048, Jiangsu, China*

Email address: [300519@njucm.edu.cn](mailto:300519@njucm.edu.cn) (L. Chen); [fenwuyi@163.com](mailto:fenwuyi@163.com) (S. Chang); [20210826@njucm.edu.cn](mailto:20210826@njucm.edu.cn) (L. Zhao); [njpibingfengli@163.com](mailto:njpibingfengli@163.com) (B. Li); [zhangsci@njucm.edu.cn](mailto:zhangsci@njucm.edu.cn) (S. Zhang); [1338200116@163.com](mailto:1338200116@163.com) (C. Yun); [xwu@njucm.edu.cn](mailto:xwu@njucm.edu.cn) (X. Wu); [mengjingyi2000@163.com](mailto:mengjingyi2000@163.com) (J. Meng); [lishiyi0803@163.com](mailto:lishiyi0803@163.com) (G. Li); [guosheng@njucm.edu.cn](mailto:guosheng@njucm.edu.cn) (S. Guo); [dja@njucm.edu.cn](mailto:dja@njutcm.edu.cn) (J. Duan)

^#^ Liangliang Chen and Siyuan Chang contributed equally to this work.

*Corresponding author.

Email: [zhangsci@njucm.edu.cn](mailto:zhangsci@njucm.edu.cn) (S. Zhang), [xwu@njucm.edu.cn](mailto:xwu@njucm.edu.cn) (X. Wu)

**Figures**

**Figure S1** UPLC analysis of the purified luteolin derivatives

(A) product 1, retention time=5.217 min; (B) product 2, retention time=5.505 min; (C) product 3, retention time=5.587 min

**Figure S2** UPLC-QToF/MS analysis of product 1

**Figure S3** ^1^H NMR spectrum of 7-SGL

**Figure S4** ^13^C NMR spectrum of 7-SGL

**Figure S5** HMBC NMR spectrum of 7-SGL

**Figure S6** HSQC NMR spectrum of 7-SGL

**Figure S7** Standard curve for determination of water solubility of luteolin

**Figure S8** Standard curve for determination of water solubility of luteoloside

**Figure S9** Standard curve for determination of water solubility of 7-SGL

**Figure S10** Water solubility of 7-SGL at different temperatures

**Tables**

**Table S1.** Solubility determination of luteolin at 30°C

**Table S2.** Solubility determination of luteoloside at 30°C

**Table S3.** Solubility determination of 7-SGL at different temperatures

(A)

(B)

(C)

**Figure S1**

**
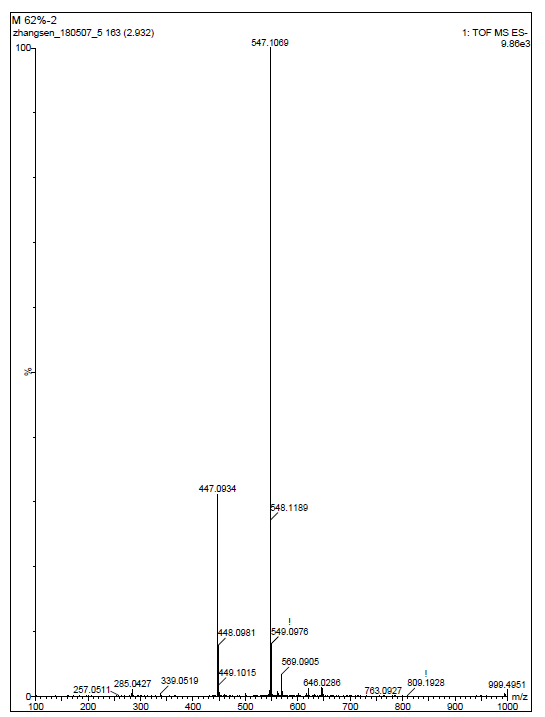
**

[M-H]^-^

product 1

**Figure S2**

**
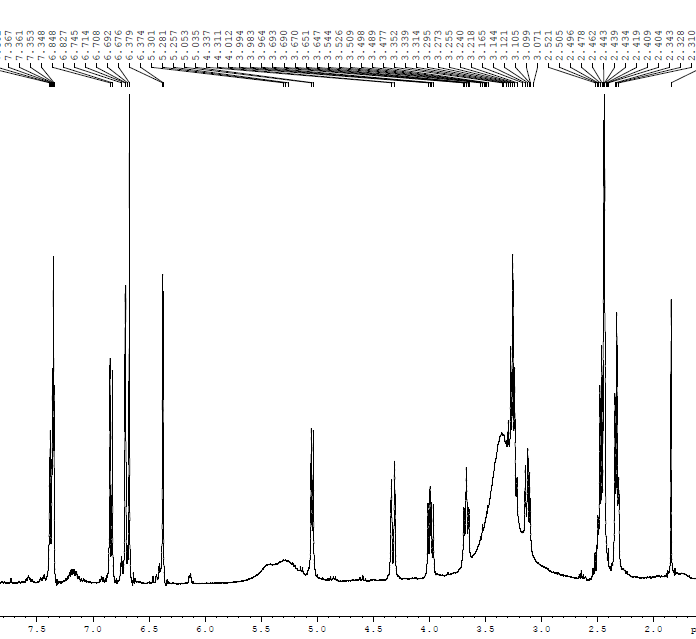
**

**Figure S3**

^1^H-NMR (DMSO-d_6_, 400 MHz) δ: 7.32-7.41 (2H, m, 2’ and 6’-H), 6.84 (1H, d, J=8.4Hz, 5’-H), 6.71 (1H, d, J=2.4Hz, 8-H), 6.68 (1H, s, 3-H), 6.38 (1H, d, J=2Hz, 6-H), 5.04 (1H, d, J=7.2Hz, 1”-H), 4.32 (1H, d, J=10.4Hz, 6”-H_A_), 3.95-4.14 (1H, m, 6”-H_B_), 3.62-3.73 (1H, m, 2’-H), 3.18-3.30 (2H, m, 3” and 5”-H), 3.05-3.15 (1H, m, 4”-H), 2.46-2.55 (2H, m, 2” ’-H), 2.30-2.38 (2H, m, 3” ’-H).

**
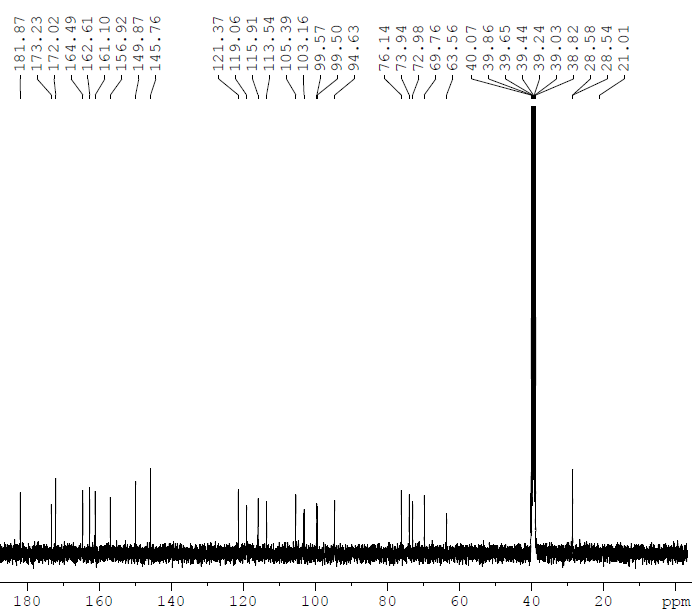
**

**Figure S4**

^13^C-NMR (DMSO-d_6_,400 MHz) δ: 181.9 (C-4), 173.2 (C-4”), 172.0 (C-1” ’), 164.5 (C-2), 162.6 (C-7), 161.1 (C-5), 156.9 (C-9), 149.9 (C-4’), 145.8 (C-3’), 121.4 (C-1’), 119.1 (C-6’), 115.9 (C-5’), 113.5 (C-2’), 105.4 (C-10), 103.2 (C-3), 99.6 (C-1”), 99.5 (C-6), 94.6 (C-8), 76.1 (C-3”), 73.9 (C-2”), 73.0 (C-5”), 69.8 (C-4”), 63.6 (C-6" ), 28.5 (C-2” ’, C-3” ’).

**
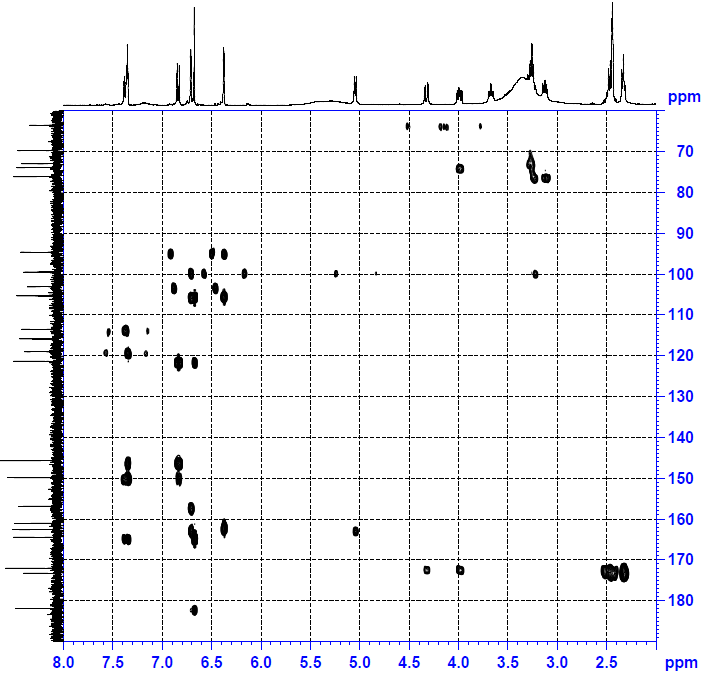
**

**Figure S5**


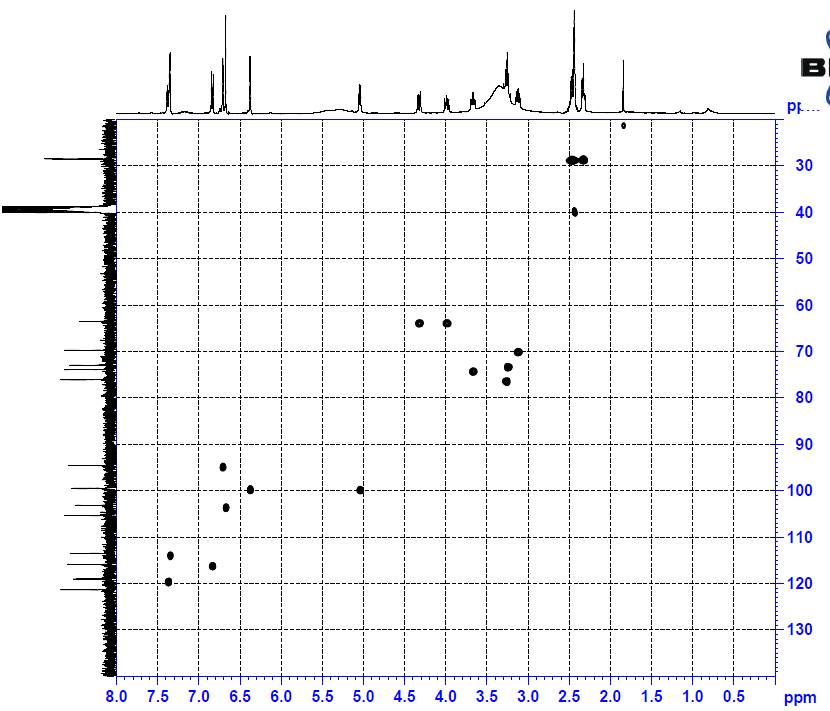


**Figure S6**


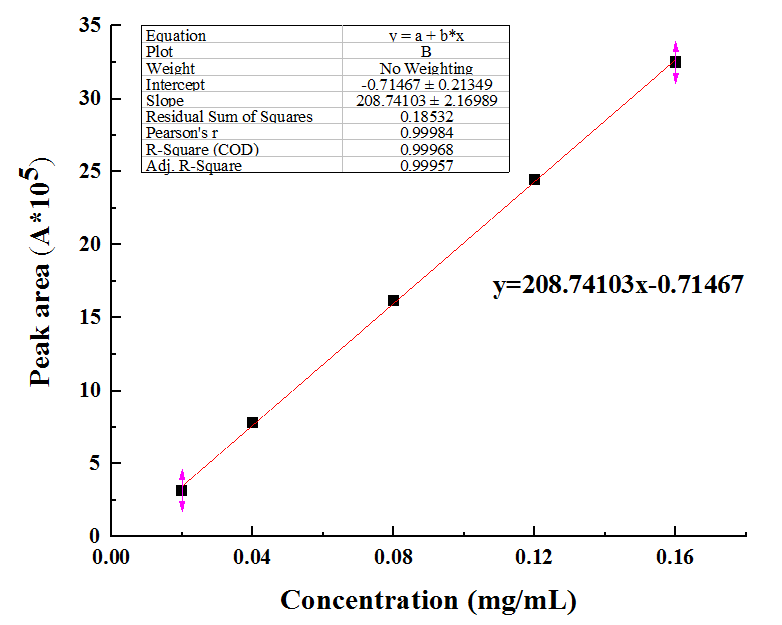


**Figure S7**


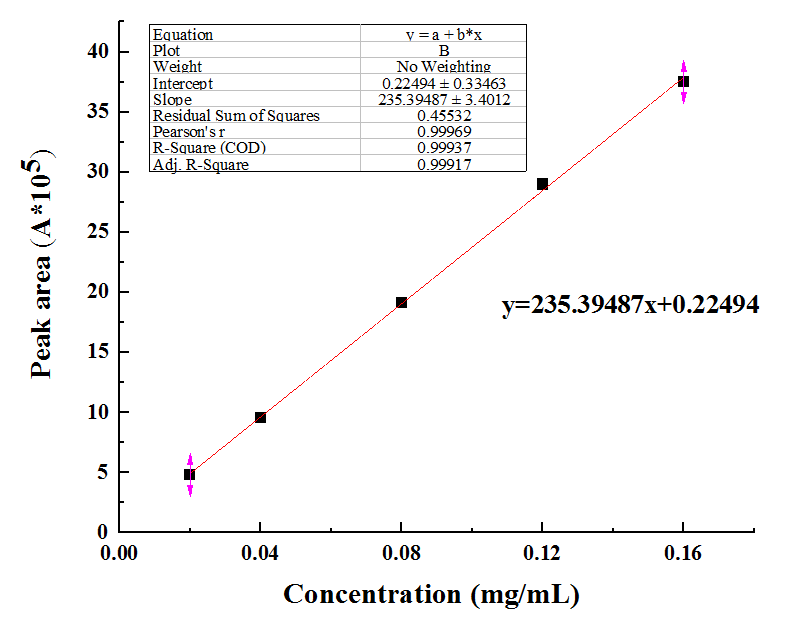


**Figure S8**


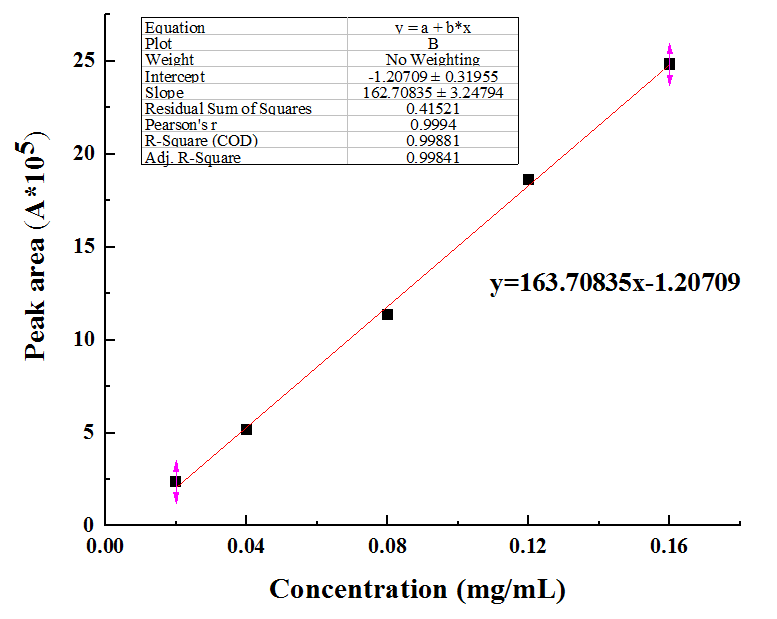


**Figure S9**


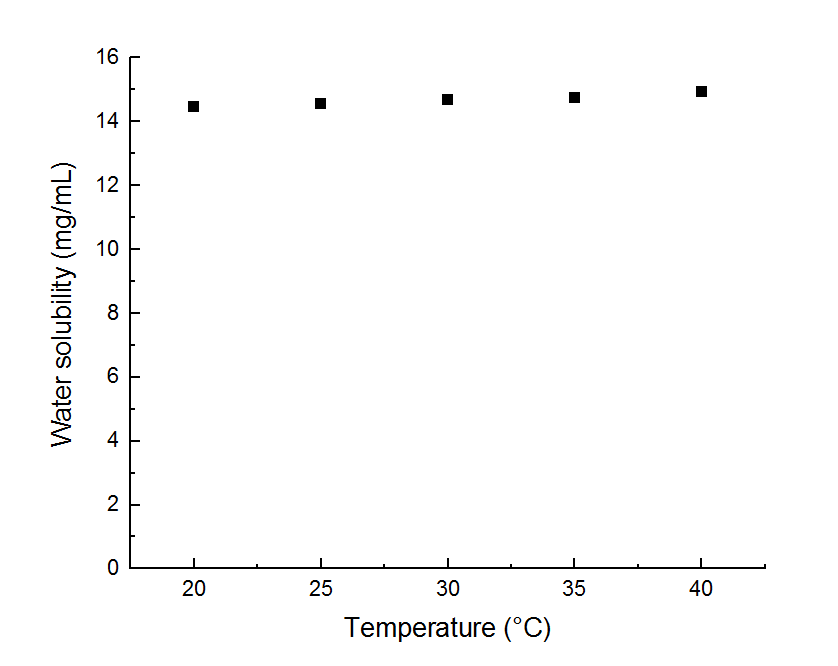


**Figure S10**

**Table S1.** Solubility determination of luteolin at 30°C

| Number | Sample | Peak area | Water solubility (mg/mL) |
| --- | --- | --- | --- |
| 1 | 0.02 g/L luteolin methanol solution | 3.1770×10^5^ | - |
| 2 | 0.04 g/L luteolin methanol solution | 7.8131×10^5^ | - |
| 3 | 0.08 g/L luteolin methanol solution | 16.1592×10^5^ | - |
| 4 | 0.12 g/L luteolin methanol solution | 24.4418×10^5^ | - |
| 5 | 0.16 g/L luteolin methanol solution | 32.5068×10^5^ | - |
| 6 | Supernatant of luteolin supersaturated aqueous solution | 12.6351×10^5^ | 0.0064 |

**Table S2.** Solubility determination of luteoloside at 30°C

| Number | Sample | Peak area | Water solubility (mg/mL) |
| --- | --- | --- | --- |
| 1 | 0.02 g/L luteoloside methanol solution | 4.8083×10^5^ | - |
| 2 | 0.04 g/L luteoloside methanol solution | 9.5465×10^5^ | - |
| 3 | 0.08 g/L luteoloside methanol solution | 19.1595×10^5^ | - |
| 4 | 0.12 g/L luteoloside methanol solution | 28.9850×10^5^ | - |
| 5 | 0.16 g/L l luteoloside methanol solution | 37.4913×10^5^ | - |
| 6 | Supernatant of l luteoloside supersaturated aqueous solution | 27.3340×10^5^ | 0.0012 |

**Table S3.** Solubility determination of 7-SGL at different temperatures

| Number | Sample | Peak area | Water solubility (mg/mL) |
| --- | --- | --- | --- |
| 1 | 0.02 g/L 7-SGL methanol solution | 2.3495×10^5^ | - |
| 2 | 0.04 g/L 7-SGL methanol solution | 5.1629×10^5^ | - |
| 3 | 0.08 g/L 7-SGL methanol solution | 11.3417×10^5^ | - |
| 4 | 0.12 g/L 7-SGL methanol solution | 18.6104×10^5^ | - |
| 5 | 0.16 g/L 7-SGL methanol solution | 24.8376×10^5^ | - |
| 6 | Supernatant of 7-SGL supersaturated aqueous solution (20°C) | 22.4619×10^5^ | 14.4580 |
| 7 | Supernatant of 7-SGL supersaturated aqueous solution (25°C) | 22.6245×10^5^ | 14.5573 |
| 8 | Supernatant of 7-SGL supersaturated aqueous solution (30°C) | 22.6758×10^5^ | 14.6782 |
| 9 | Supernatant of 7-SGL supersaturated aqueous solution (35°C) | 22.9386×10^5^ | 14.7492 |
| 10 | Supernatant of 7-SGL supersaturated aqueous solution (40°C) | 23.2259×10^5^ | 14.9247 |
